# The Microbiome-Mitochondrion Connection: Common Ancestries, Common Mechanisms, Common Goals

**DOI:** 10.1128/mSystems.00018-17

**Published:** 2017-05-09

**Authors:** Alfredo Franco-Obregón, Jack A. Gilbert

**Affiliations:** a Department of Surgery, National University of Singapore, Singapore; b BioIonic Currents Electromagnetic Pulsing Systems Laboratory, BICEPS, National University of Singapore, Singapore; c Bioscience Division, Argonne National Laboratory, Argonne, Illinois, USA; d Department of Surgery, The Microbiome Center, University of Chicago, Chicago, Illinois, USA; e The Microbiome Center, Marine Biological Laboratory, Woods Hole, Massachusetts, USA; University of Hawaii at Manoa

**Keywords:** SCFA, butyrate, ellagitannins, lactate, metabolic, microbiome, mitochondria, muscle, short-chain fatty acids, urolithin A

## Abstract

Lynn Margulis in the 1960s elegantly proposed a shared phylogenetic history between bacteria and mitochondria; this relationship has since become a cornerstone of modern cellular biology. Yet, an interesting facet of the interaction between the microbiome and mitochondria has been mostly ignored, that of the systems biology relationship that underpins host health and longevity. The mitochondria are descendants of primordial aerobic pleomorphic bacteria (likely genus *Rickettsia*) that entered (literally and functionally) into a mutualistic partnership with ancient anaerobic microbes (likely *Archaea*). A stable symbiosis was established, given the metabolic versatility of the early mitochondria, which were capable of providing energy with or without oxygen, whereas nutrient gathering was the assumed responsibility of the host. While microbial relationships with single-cell protists must have occurred in the past, as they occur today, the evolution of multicellular organisms generated a new framework for symbiosis with the microbial world, taking the ancient partnership to an entirely new level. Cell-cell communication between microbes and single-cell protists was augmented through multicellularity to allow distant communication between the host cells and the microbiome, resulting in the development of complex metabolic relationships and an immune system to manage these interactions. Thus, the host is now the body and its resident mitochondria, and the microbiome is an essential supplier of metabolites that act at the level of mitochondria in skeletal muscle to stabilize host metabolism. We humans are caretakers of a profoundly vast and diverse microbiota, the majority of which resides in the gut. Indeed, the microbial genetic diversity of our microbiota outstrips our own by several orders of magnitude, and the cellular abundance is roughly equivalent to our somatic selves. Modern clinical science has elegantly highlighted the importance of the microbiome for metabolic health and well-being. This perspective underscores one fundamental facet of this symbiosis, the ancestral mitochondrion-microbiome axis.

## Abstract (precis)

Lynn Margulis in the 1960s elegantly proposed a shared phylogenetic history between bacteria and mitochondria; this relationship has since become a cornerstone of modern cellular biology. Yet, an interesting facet of the interaction between the microbiome and mitochondria has been mostly ignored, that of the systems biology relationship that underpins host health and longevity.

## PERSPECTIVE

Nutrient metabolism is a function shared by both the microbiome and mitochondria. In recent years, it has become increasingly evident that the gut microbiome produces metabolites that influence mitochondrial function and biogenesis (i.e., mitochondrial replication within a cell to increase ATP production). Recent studies have highlighted the importance of three key microbiome metabolites: (i) the short-chain fatty acids (SCFA), (ii) the urolithins, and (iii) lactate. In particular, among these key metabolites is the SCFA butyrate, which is produced by microbial fermentation of indigestible fiber by a number of different bacterial lineages, including the *Clostridium* and *Butyrivibrio* genera (1–3), and urolithin A, which is produced by lactobacilli and *Bifidobacterium* from ellagitannins present in certain fruits, berries, and nuts (4, 5). Both urolithin A and butyrate have been shown to enhance microbial diversity, as well as promoting the abundance of bacteria that generate these compounds (5). In parallel, SCFAs are known to activate AMP kinase, which might serve to induce mitochondriogenesis, revealing an alternative systemic limb of the interrelationship (6). As a mitochondrial energy source, butyrate is able to rescue respiratory depression of colonocytes in germfree mice (3). It is thus not surprising that the same bacteria that generate these compounds are also able to confer resistance to metabolic disturbance (6). A link between SCFA-producing and urolithin A-producing bacteria exists in lactic acid metabolism, whereby the lactic acid produced by lactobacilli and bifidobacteria (and possibly mitochondria) supports the production of butyrate from SCFA-producing microbes (e.g., clostridia), accompanied by the production of ATP (7–9). There are therefore explicit and implicit interactions between lactobacilli, bifidobacteria, SCFA-producing bacterial strains, and the metabolites they produce that have broad systemic metabolic ramifications if the correct tissues are targeted.

Metabolic health and mitochondrial health are synonymous. Mitochondria are our predominant site of substrate oxidation. Muscle represents, proportionately, our largest tissue mass and hence our greatest unified site of mitochondria, as well as one of our most metabolically active tissues. Both mitochondrion and muscle functions are positively impacted by physical activity and diet. An obvious systems biology link couples muscle health to microbial activity, and vice versa. On the one hand, the host’s fitness level correlates with higher fecal butyrate levels (2, 7, 10) as well as an increase in the fecal concentration of *Clostridium* spp. and lactobacilli (6, 11–14). On the other hand, both butyrate (15) and urolithin A (16, 17) enhance skeletal muscle’s oxidative capacity and mitochondrial function. These results corroborate a muscle-microbiome reinforcement from either direction.

Of particular interest, probiotic supplementation with *Lactobacillus plantarum* (TWK10) exerts potent effects over muscle performance and oxidative capacity (8) as well as an increase in colonic SCFA content (10). Skeletal muscle activity, via its capacity to release myokines that quench systemic inflammation, increase fatty acid oxidation, and promote mitochondriogenesis, also promotes microbiome diversity (6, 18). Butyrate and urolithin A may thus act synergistically at the level of the mitochondrion and by mere virtue of muscle’s sheer predominance preferentially influence muscle function and enhancement of systemic metabolism, strengthening the muscle-microbiome bond. Although speculative, existing evidence also links muscular lactic acid production to microbiome function, possibly even extending to components of the microbiome outside the gut to reinforce and broaden this dynamic interaction. Indeed, an increased relative abundance of lactobacilli (6, 12–14) and *Clostridiaceae* following exercise is positively correlated with blood lactate accumulation, which reflects fitness levels (14, 19). These results raise the provocative possibility of targeting muscle for systemic metabolic improvement via specific nutritional intervention aimed at the microbiome.

The ability of specified microbiome lactobacilli to metabolize polyphenols found in certain fruits (16) or fermented soy (8), which work in concert with SCFAs such as butyrate (10, 20) to enhance muscle oxidative capacity by stimulating mitochondria, can potentially be exploited to produce a form of metabolic stabilization somewhat reminiscent of that exerted by physical activity. Finally, as advanced age is associated with both muscle loss (sarcopenia) and microbiome dybiosis, such a therapeutic approach holds the potential of slowing the onset of several metabolic and structural deficits inflicted by aging in elderly people.

The potential for precise therapeutic interventions that target microbial-mitochondrial metabolic communication provides a novel avenue for the treatment of many metabolic disturbances and could have profound implications for the future of medical treatments. From an ancient union, the dominance of the microbial world is redefining our perspective for health and wellness.
